# Comparative transcriptomic analysis of gonadal development and renewal in the ovoviviparous black rockfish (*Sebastes schlegelii*)

**DOI:** 10.1186/s12864-021-08169-x

**Published:** 2021-12-04

**Authors:** Jianshuang Li, Likang Lyu, Haishen Wen, Yun Li, Xiaojie Wang, Ying Zhang, Yijia Yao, Xin Qi

**Affiliations:** 1 College of Fishery, Ocean University of China, Qingdao, 266000 P. R. China; 2 Key Laboratory of Mariculture, Ocean University of China, Ministry of Education, Qingdao, 266003 P. R. China

**Keywords:** Black rockfish, Transcriptomic, Gonadal development, RNA-seq

## Abstract

**Background:**

The black rockfish (*Sebastes schlegelii*) has an ovoviviparous reproductive pattern and long-term sperm storage, resulting in asynchronous gonadal development between the sexes. However, the comprehensive understanding of gonadal development in black rockfish has not yet been achieved. Here, we studied gonadal development and germ cell renewal using histology and RNA-seq.

**Results:**

In this study, RNA-seq was performed on testes and ovaries to characterize key pathways and genes that are active during development and gamete maturation in black rockfish. Differentially expressed genes (DEGs) were identified and annotated in 4 comparisons (F_III vs. F_IV, F_IV vs. F_V, M_III vs. M_IV and M_IV vs. M_V). Based on analysis of DEGs enriched in the testis, 11 and 14 Kyoto Encyclopedia of Genes and Genomes (KEGG) pathways were mapped to the M_III vs. M_IV group and the M_IV vs. M_V group, respectively. DEGs in ovarian development were also classified into 10 groups according to their biological functions. The expression patterns of the selected genes determined by qPCR were significantly correlated with the RNA-Seq results, supporting the reliability and accuracy of the RNA-Seq analysis. E_2_ levels showed down regulation from previtellogenesis to mature stage in female and T level showed down regulation from spermatogenesis to regressed stage in the male.

**Conclusions:**

The categories “intercellular interaction and cytoskeleton”, “molecule amplification” and “repair in the cell cycle” were revealed to be crucial in testis development and spermatogenesis, as was the biosynthesis of a series of metabolites. Our results provide comprehensive insight into black rockfish gonadal development and provide a basis for further study of reproductive physiology and molecular biology in ovoviviparity teleosts.

**Supplementary Information:**

The online version contains supplementary material available at 10.1186/s12864-021-08169-x.

## Background

Spermatozoa and oocytes in fish species are highly specialized cells that fuse into an embryo that will develop into a mature organism that produces gametes [1]. Germ cell development and renewal are required for gonadal development during the entire reproductive lifespan of most teleosts. Spermatogenesis and oogenesis are both highly organized processes and are crucial for transmitting genetic information to the next generation [2, 3]. Although the development of spermatozoa and oocytes follows common principles, there are still many differences between females and males in terms of gametogenesis [2]. Teleosts represent the most diverse and numerous groups of vertebrates. Research on spermatogenesis in teleosts has mainly focused on a few species, such as Atlantic cod (*Gadus morhua*) [4], tilapia (*Oreochromis niloticus*) [5], rainbow trout (*Oncorhynchus mykiss*) [6, 7], zebrafish (*Danio rerio*) [8], European eel (*Anguilla anguilla*) [9], and Atlantic salmon (*Salmo salar*) [10]. The molecular mechanism of spermatogenesis in fishes has also been reviewed [11]. In addition, studies of oogenesis in teleost fishes have been reported in recent decades, including the related genes in tilapia [5], rainbow trout [12], least killifish (*Heterandria formosa*) [13], pejerrey (*Odontesthes bonariensis*) [14] and Atlantic molly (*Poecilia mexicana*) [15].

In recent years, molecular techniques have increased in importance as tools for the identification of key pathways and genes involved in biological processes. RNA-seq platforms based on next-generation sequencing technology (NGS) provide a revolutionary and efficient tool for investigating the key pathways and genes in gonadal development. Such platforms have already been applied to the study of the reproductive system in many species, including pig (*Sus scrofa domesticus*) [16], American alligator (*Alligator mississippiensis*) [17], and swimming crab (*Portunus trituberculatus*) [18]. In teleosts, an increasing number of studies have focused on gonadal development and germ cell renewal. In a study of tilapia (*Oreochromis niloticus*), RNA-seq was used to reveal the genetic framework underlying sex determination and sexual differentiation [19]. The Y chromosome sequence identified BCAR1 as a potential sex determination gene in channel catfish (*Ictalurus punctatus*) [20]. In addition, many studies have focused on gonadal development and germ cell renewal in teleosts, revealing the potential mechanism of germ cell and gonadal development [21, 22] and identifying sex-related genes in both sexes [23, 24].

Reproductive strategy represents one of the most critical traits in life due to its effects on fitness and survival [25]. Fishes adopt various reproductive strategies, including oviparity, in which eggs are laid before fertilization (a common reproductive strategy in teleosts), viviparity, in which embryos develop inside ovaries while receiving nutrition directly from the mother (employed in some Chondrichthyes species) [26], and the recently well-studied ovoviviparity, in which eggs are fertilized in ovaries with the development of lecithotrophic larvae (this strategy is used by some teleosts, including black rockfish (*Sebastes schlegelii*) [27–29]). Black rockfish undergo nonsynchronous gonadal development. Spermatogenesis begins in July and is completed in November and December, and then, mating occurs through a modified urogenital papilla, which emits spermatozoa into the female ovary. The sperm are stored in the ovary during vitellogenesis and under the ovigerous lamellae epithelium during the late period [27]. In females, oocytes begin vitellogenesis in November and mature in late March. After the activation of sperm and fertilization in April, the females become pregnant, and the fertilized eggs develop into larvae in the ovary until parturition occurs in May [30, 31]. The asynchrony of gonadal development in black rockfish could be responsible for the ovoviviparous reproductive strategies.

Previous studies of black rockfish have mainly focused on environmental toxicology and immunity [32–34], response to stress [28, 35], whole genomic data analysis [36, 37] and reproductive physiology [38–41]. Most studies of reproduction in black rockfish have focused on single gene identification [38–40, 42, 43] and analysis of gene function [44, 45]. However, a comprehensive understanding of gonadal development in black rockfish is lacking. In this study, RNA-seq was performed on the testes and ovaries of black rockfish to characterize key pathways and genes that are active during development and gamete maturation in this species. The transcripts were *de novo* assembled and annotated, greatly enriching the black rockfish gene database. The pathways and genes identified in this study provide novel insight into the reproductive biology of ovoviviparity teleosts.

## Results

### Identification of the stages of development of the ovary and testis through collection of basic physiological data and histological analysis

**Fig. 1:**
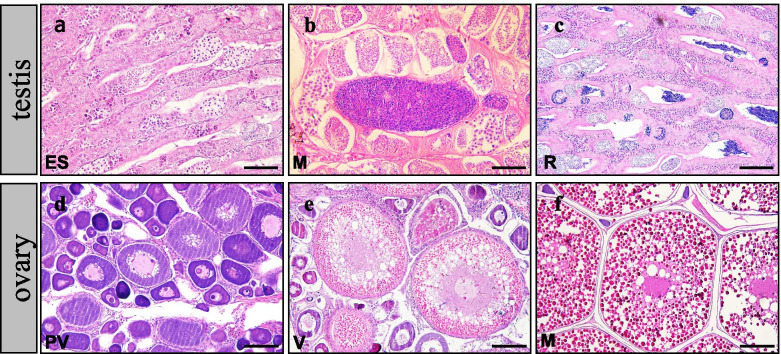
H&E-stained sections illustrating the gonadal development stages of the black rockfish. ES: early spermatogenesis; M: mature; R: regressed; PV: previtellogenesis; V: vitellogenesis. Scale bars (**a**, **b**, **c**) = 50 μm. Scale bars (**d**, **e**) = 100 μm. Scale bars (**f**) = 200 μm

H&E staining was performed to identify the different stages of gonadal development that occur from October to March of the next year. As shown in Fig. 1, the testis was found to be in an early spermatogenesis stage (ES) from October until early November, when spermatogenesis was completed, marking the mature stage (M). After mating in December, sperm were totally emptied from the testis, showing that the testis was in the regressed stage (R). Previtellogenesis stage (PV) oocytes with lipid droplets and yolk accumulation initiation were observed in early November. Lipid droplets and yolks were observed to fuse in oocytes at the vitellogenesis stage (V). Completion of the first meiotic division and first polar body discharge were observed in mature-stage oocytes (M).

According to the identification by H&E staining, 3 different stages of male and female gonads were distinguished; there were termed F_III, F_IV and F_V, representing the previtellogenesis stage, the vitellogenesis stage, and the mature stage of ovarian development and M_III, M_IV and M_V, representing the early spermatogenesis stage, the mature stage, and the regressed stage in the testis. The average body weight and gonad weight of the collected samples are shown in Additional file 1. In male rockfish, both high body weight (795.09 g±58.63 g) and high gonadosomatic index (GSI) (1.26) were observed in winter, the time during which mating behavior occurs in ovoviviparous black rockfish. In females, high body weight (880.68 g±99.48 g) and GSI (7.57) were observed in mid-March when the oocytes matured and fertilization occurred.

### *de novo* assembly and annotation of black rockfish gonadal transcripts

**Fig. 2:**
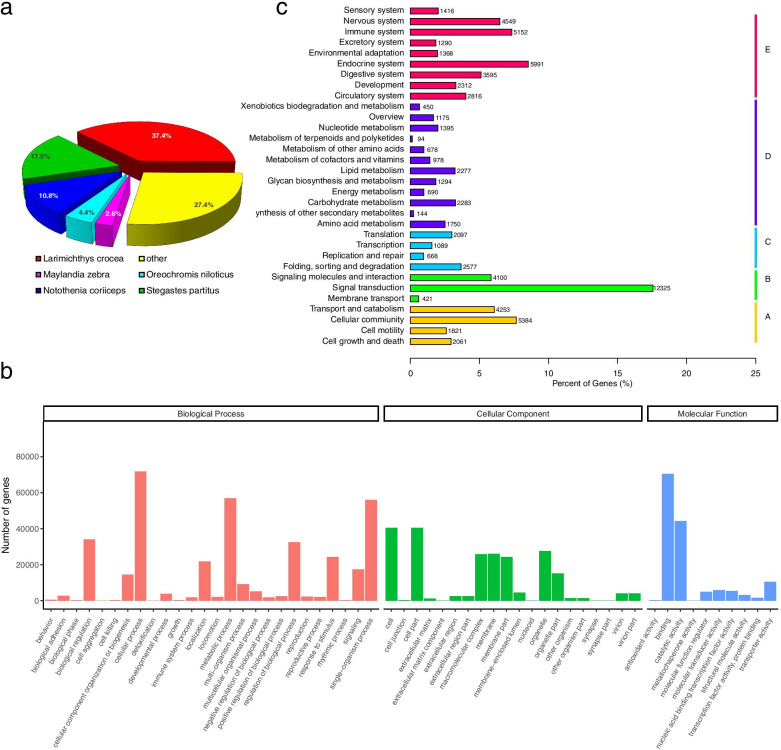
Annotation and functional classification of transcripts obtained from the gonads of black rockfish. (**a**) Top-hit species distribution of BLASTX matches of assembled transcripts. (**b**) Functional annotation of assembled transcripts based on gene ontology (GO) analysis. GO analysis was performed at level 2 for the three main categories (biological process, cellular component, and molecular function). The x-axis shows the specific terms. The y-axis shows the number of transcripts for each term. (**c**) Pathway assignment based on the Kyoto Encyclopedia of Genes and Genomes (KEGG) database. The transcripts were classified into five main categories (A: cellular process, B: environmental information processing, C: genetic information processing, D: metabolism and E: organismal systems)

RNA-Seq was performed on ovary and testis samples from 3 different stages. A total of 1,029,619,820 raw reads (150 bp) were obtained from 18 gonad samples using the Illumina HiSeq X Ten platform. After preprocessing and filtration of low-quality sequences, the clean read count was 998,950,272 (Additional file 2). The *de novo* assembled transcriptome included 517,848 transcripts with an N50 of 1,660, indicating a high-quality assembly. The transcripts of black rockfish gonads were annotated in 7 public databases, including Nr, Nt, KO, SwissProt, Pfam, Gene Ontology (GO) and KOG with 61.63 % of genes annotated in at least 1 database. The nonredundant (NR) annotation showed that 72.6 % of transcripts were annotated in 5 fish species, with the highest sequence similarity to large yellow croaker (*Larimichthys crocea*) (Fig. 2a).

The functional classification of transcriptome data is a basic requirement for the application of functional genomic approaches in fishery research. Analyses based on the GO and KEGG databases are commonly used in the functional classification of transcriptomic sequences. The results obtained for our transcriptome data showed that Blast2Go assigned 123,181 transcripts to 56 functional GO terms (Fig. 2b). Regarding the 3 primary ontology categories, the majority of the annotations (26 terms) fell into the category of “biological process” (BP), followed by “cell component” (CC) (20 terms) and “molecular function” (MF) (10 terms). Based on the analysis of level 2 GO terms, “cellular process” (GO:0009987) showed the most annotated genes in BP. For CC, “cell” (GO:0005623) and “cell part” (GO:0044464) contained the highest numbers of annotations. The GO terms related to MF with the highest number of annotations were “binding” (GO:0005488) and “catalytic activity” (GO:0003824). To understand the higher-order functional information associated with the biological system, KEGG analysis was performed [46]. Based on the analysis, 70,174 genes were annotated into 5 categories that included 32 significantly enriched KEGG pathways (Fig. 2c).

### Analysis of genes that are differentially expressed at various gonadal development stages

**Fig. 3:**
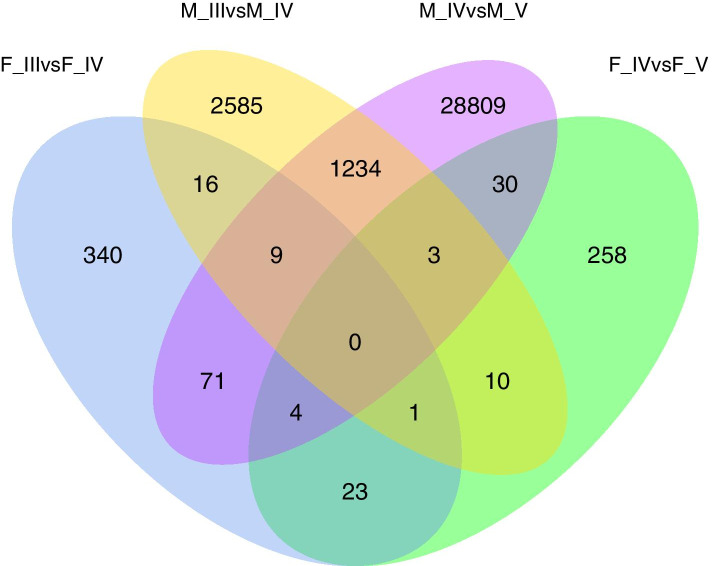
Venn diagram of the gonadal DEGs identified in 4 comparisons in black rockfish

A total of 33,393 DEGs were obtained from 4 different gonadal development libraries (adjusted *p value*<0.01 and absolute log_2_ fold change>2) (Fig. 3). Of these, 464 DEGs (151 upregulated and 313 downregulated) were significantly differentially expressed in F_III vs. F_IV, and 329 DEGs (109 upregulated and 220 downregulated) were significantly differentially expressed in F_IV vs. F_V. Although males spent less time in the reproductive period than females, many more DEGs were found in males than in females, of these DEGs, 3,858 (1,611 upregulated and 2,247 downregulated) were significantly differentially expressed in M_III vs. M_IV, and 30,160 DEGs (24,446 upregulated and 5,714 downregulated) were significantly differentially expressed in M_IV vs. M_V.

### Identification of DEGs during testis development in black rockfish

**Fig. 4:**
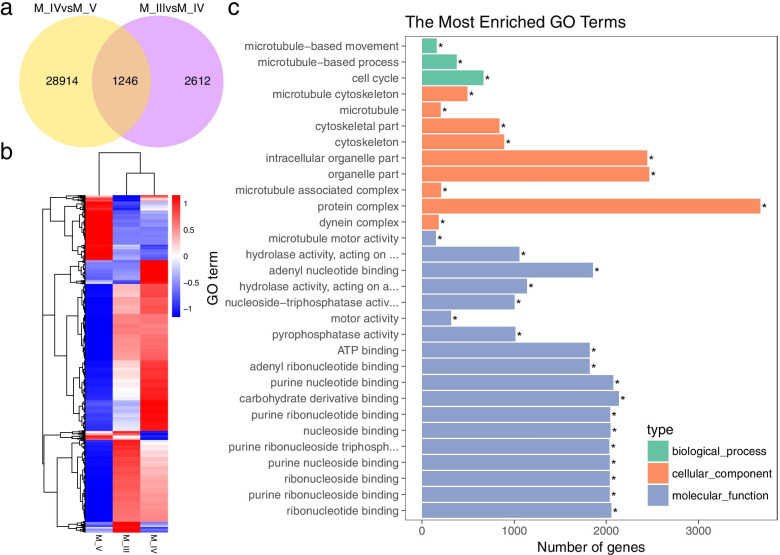
Identification and annotation of DEGs at different developmental stages of the testes in black rockfish. (**a**) Venn diagram of the DEGs of M_III vs. M_IV and M_IV vs. M_V. (**b**) The expression values of 32,772 DEGs in 3 libraries (M_III, M_IV, M_V) are presented in a heat map. Red and blue colors indicate up- and downregulated transcripts, respectively. (**c**) Gene ontology (GO) analysis of the 32,772 DEGs. The x-axis shows the number of genes in each term. The y-axis shows the specific terms. The asterisk represents the corrected *p value* <0.05 for each GO term

Based on the DEGs mentioned above, 3,858 and 30,160 annotated DEGs were obtained from the M_III vs. M_IV group and from the M_IV vs. M_V group, respectively (Fig. 4a). Heatmap analysis of the 32,772 DEGs expressed at all 3 stages of testis development revealed that most of the genes in both early spermatogenesis stage and mature stage testis showed similar expression pattern, which upregulated genes at the early spermatogenesis stage (M_III) and the mature stage (M_IV) of the testis were downregulated at the regressed stage (M_V) of the testis, suggesting that the expression profiles of these DEGs and the male reproductive process, including gamete maturation, were directly proportional (Fig. 4b). GO analysis of these DEGs showed that most of them mapped to MF terms (*p v*alue<0.01), especially to GO terms related to molecule binding (“anion binding”, GO:0043168, “small molecule binding”, GO:0036094 and others) (Fig. 4c).

**Fig. 5:**
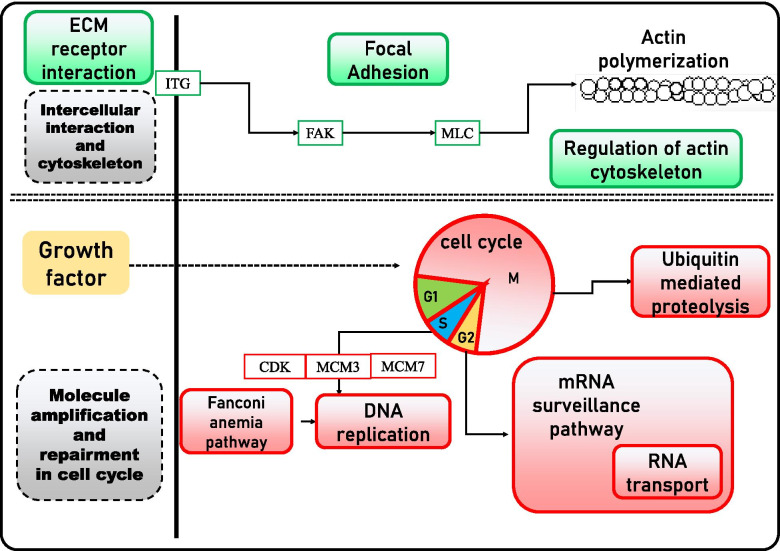
Regulation of pathways in the M_III vs. M_IV group of black rockfish. Red and green orthogons represent up- and downregulated KEGG pathways and DEGs, respectively [46, 86, 90]

Eleven and 14 KEGG pathways were significantly differentially enriched between the M_III vs. M_IV group and between the M_IV vs. M_V group, respectively (*p value* <0.01) (Table 1). In the M_III vs. M_IV group, 11 KEGG pathways were classified into 3 categories, including “intercellular interaction and cytoskeleton”, “molecule amplification and repairment in the cell cycle”, and “other” (Fig. 5). The DEGs in the category intercellular interaction and cytoskeleton, including “extracellular matrix (ECM)-receptor interaction”, “focal adhesion”, and “regulation of actin cytoskeleton”, were upregulated in the mature stage compared with the early spermatogenesis stage. DEGs in the category molecular amplification and repairment in the cell cycle, including the “cell cycle”, “ubiquitin-mediated proteolysis”, “DNA replication”, “Fanconi anemia”, “RNA transport” and “mRNA surveillance” pathways, were significantly enhanced in the early spermatogenesis stage, when spermatogenesis began and cell division and protein biosynthesis proceeded, compared with the mature stage. Some other pathways, such as steroid hormone biosynthesis, were also upregulated in mature-stage testes.

**Fig. 6:**
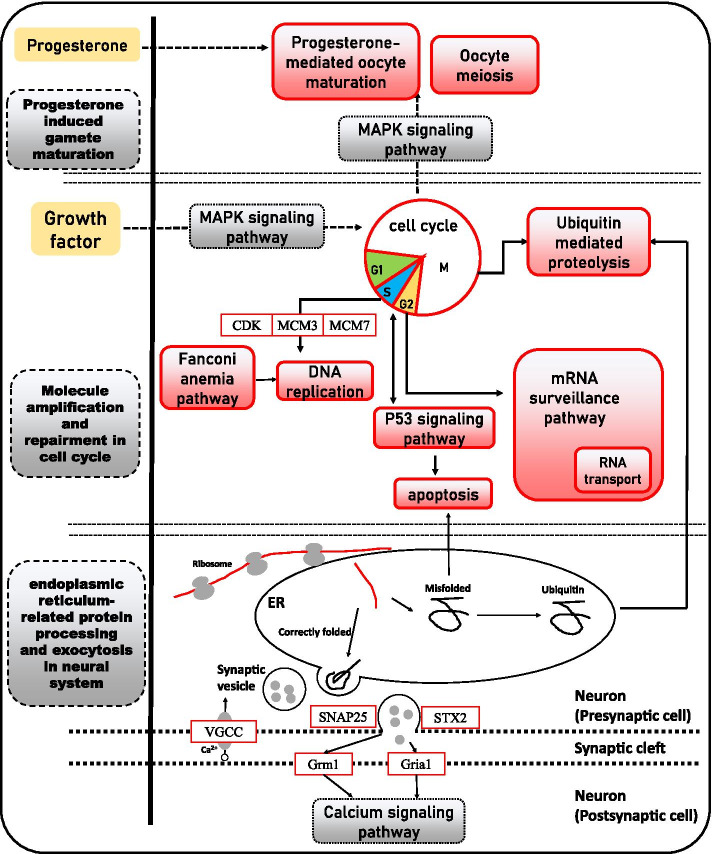
Regulation of pathways in the M_IV vs. M_V group of black rockfish. Red and green orthogons represent up- and downregulated KEGG pathways and DEGs, respectively [46, 86, 90]

As shown in Fig. 6, the 14 KEGG pathways associated with the DEGs in the M_IV vs. M_V groups were classified into 5 categories, including “progesterone-induced gamete maturation”, “molecule amplification and repairment in the cell cycle”, “endoplasmic reticulum-related protein processing”, “exocytosis in the nervous system”, and “infection and immune-related”. In the category progesterone-induced gamete maturation, most DEGs were upregulated in early spermatogenesis stage testes due to the importance of steroid hormones in germ cell division. The category molecule amplification and repairment in the cell cycle was also upregulated both at the early spermatogenesis stage and at the mature stage, implying that marked changes in cellular metabolism continue throughout the entire reproductive process. “Protein processing in the endoplasmic reticulum”, “synaptic vesicle cycle” and “retrograde endocannabinoid signaling” in the categories endoplasmic reticulum-related protein processing and exocytosis in the nervous system showed the most interesting and abundant DEGs. DEGs such as *sar1b*, *Sect. 13*, *sec24c* and *slc18a2* are involved in transport via vesicles, *stx2* is related to epithelial morphogenesis due to exocytosis, *cacna1b* regulates hormone and neurotransmitter release, and *gria1* and *grm1* receive messages transmitted through the neurotransmitter glutamate. It is suggested that the genes that are differentially expressed in the early spermatogenesis stage and the mature stage are inseparable from the intense reproductive process.

As shown in Additional file 3, 3 KEGG pathways, “cell cycle”, “mRNA surveillance” and “RNA transport”, were upregulated in both M_III vs. M_IV and M_IV vs. M_V, indicating that the activity process occurred throughout the period from early spermatogenesis to regression in the testis. Interestingly, ubiquitin-mediated proteolysis, which was highly expressed in M_III vs. M_IV and M_IV vs. M_V, presented totally different DEGs, suggesting that this pathway may play different roles at different developmental stages. In the oocyte meiosis pathway, *sgo1*, *stag3*, *smc1b* and *smc3* were upregulated at the mature stage, suggesting the exit of gametes from meiosis in the final reproductive stage. In addition, *ccne1*, *ccnd2*, and *cdk2*, DEGs in the p53 signaling pathway, were upregulated, and *srsf10* was downregulated. *srsf10* represses cell cycle G1 and G2 phase arrest by inhibiting *ccne1*, *ccnd2*, and *cdk2*, resulting in activation of the cell cycle in testes at the early spermatogenesis stage and the mature stage.

**Table 1:** Significantly enriched KEGG pathways in the M_IIIvsM_IV and M_IVvsM_V groups

Stage	KEGG pathway	ID	Input gene	Regulation
M_IIIvsM_IV				
Intercellular interaction and cytoskeleton	ECM-receptor interaction	ko04512	39	DOWN
	Focal adhesion	ko04510	64	DOWN
	Regulation of actin cytoskeleton	ko04810	59	DOWN
Molecule amplification and repair in the cell cycle	Cell cycle	ko04110	39	UP
	Ubiquitin-mediated proteolysis	ko04120	27	UP
	DNA replication	ko03030	10	UP
	Fanconi anemia pathway	ko03460	12	UP
	mRNA surveillance pathway	ko03015	23	UP
	Steroid hormone biosynthesis	ko00140	21	DOWN
	Glycosylphosphatidylinositol (GPI)-anchor biosynthesis	ko00563	7	UP
M_IVvsM_V				
Progesterone- induced gamete maturation	Progesterone-mediated oocyte maturation	ko04914	117	UP
	Oocyte meiosis	ko04114	129	UP
Molecule amplification and repair in the cell cycle	mRNA surveillance pathway	ko03015	97	UP
	Cell cycle	ko04110	140	UP
	Ubiquitin-mediated proteolysis	ko04120	143	UP
	RNA transport	ko03013	139	UP
	p53 signaling pathway	ko04115	69	UP
	Apoptosis - multiple species	ko04215	34	UP
Endoplasmic reticulum-related protein processing and exocytosis in nervous system	Protein processing in endoplasmic reticulum	ko04141	159	UP
	Synaptic vesicle cycle	ko04721	102	UP
	Retrograde endocannabinoid signaling	ko04723	179	UP
Infection- and immune-related	*Staphylococcus aureus* infection	ko05150	49	DOWN
	Transcriptional misregulation in cancer	ko05202	174	DOWN
	Pertussis	ko05133	73	DOWN
	RNA transport	ko03013	30	DOWN
	Huntington’s disease	ko05016	205	UP

3.2 Identification of DEGs during ovarian development in the black rockfish.

**Fig. 7:**
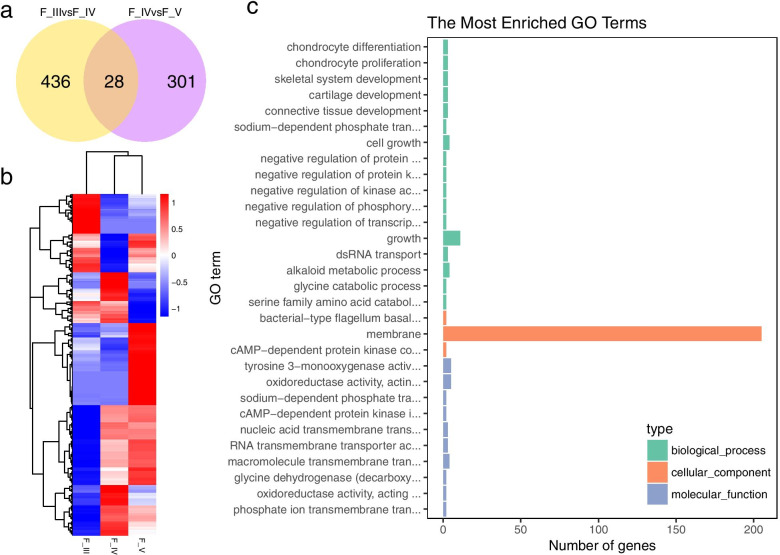
Identification and annotation of DEGs at different developmental stages in the ovaries of black rockfish. (**a**) Venn diagram of the DEGs of F_III vs. F_IV and F_IV vs. F_V. (**b**) The expression values of 765 DEGs in 3 libraries (F_III, F_IV, F_V) are presented in a heat map. Red and blue colors indicate up- and downregulated transcripts, respectively. (**c**) Gene ontology (GO) analysis of the 765 DEGs. The x-axis shows the number of genes in each term. The y-axis shows the specific terms. The asterisk represents the corrected *p value* <0.05 for each GO term

Due to the specialized reproductive strategy of female black rockfish, the transcriptomic changes in the ovary were not as marked as those in the testis. Annotation and enrichment analysis identified only 464 and 329 DEGs (adjusted *p value*<0.01 and absolute log_2_ fold change>2) in the F_III vs. F_IV group and the F_IV vs. F_V group, respectively (Fig. 7a). The expression patterns of all 765 DEGs differed from those in the testis, and these genes were upregulated in the ovary at the vitellogenesis stage (F_IV) and at the mature stage (F_V), suggesting that the development of the ovary is delayed compared with that of the testis (Fig. 7b).

Interestingly, GO analysis of these 765 DEGs resulted in annotation of a total of 205 DEGs in the membrane (GO:0016020) in CC (Fig. 7c). Additional file 4 shows detailed information on these 205 DEGs, most of which participate in membrane-anchored enzymatic reactions or molecular transport. Eleven biological function classifications and several subclassifications were identified: cell cycle, cell junction, cell structure, metabolism, DNA binding and transcription regulated, immune system, nervous system, molecular transport, protein modification, RNA binding, and signal transduction. Among these classifications, molecular transport was the most abundant (40 DEGs), indicating the importance of the material transport process during the ovarian development stage in black rockfish.

### Validation of RNA-Seq results by qPCR

**Fig. 8:**
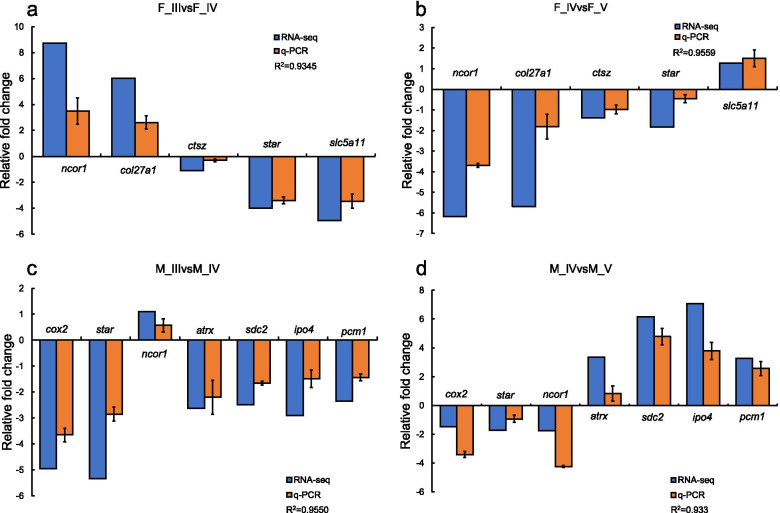
qPCR validation of 10 differentially gonadally expressed genes generated from the RNA-Seq results. The expression levels of the selected genes were normalized to the *18 S* gene. a: F_III vs. F_IV; b: F_IV vs. F_V; c: M_III vs. M_IV; d: M_IV vs. M_V. Gene abbreviations are: nuclear receptor corepressor 1 (*ncor1*); collagen alpha-1(XXVII) chain B (*col27a1*); cathepsin Z (*ctsz*); steroidogenic acute regulatory protein (*star*); sodium/myo-inositol cotransporter (*slc5a11*); cyclooxygenase 2 (*cox2*); transcriptional regulator ATRX (*atrx*); syndecan-2 (*sdc2*); importin-4 (*ipo4*); and pericentriolar material 1 protein (*pcm1*)

To validate the RNA-Seq data, ten DEGs were randomly selected and subjected to qPCR analysis. The results showed that the qPCR expression pattern of the selected genes was significantly correlated with the RNA-Seq results (R^2^: 0.933-0.9559). In total, the RNA-Seq data were confirmed by the qPCR results, implying the reliability and accuracy of the RNA-Seq analysis (Fig. 8).

### Hormone concentrations assay

**Fig. 9:**
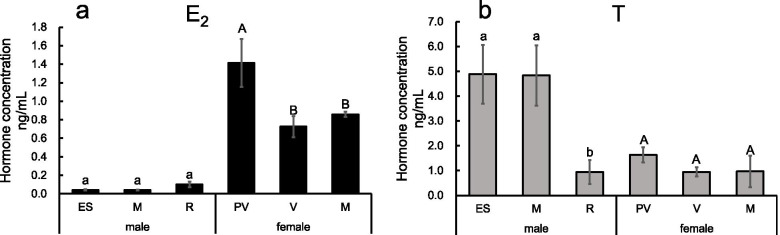
Measurement of E_2_ (**a**) and T (**b**) concentrations at different developmental stages of the ovary and testis. Four individuals were analyzed in each developmental stage. Different lowercase letters indicate significant differences between developmental stages in the testis (*p<0.05*). Different capital letters indicate significant differences between developmental stages in the ovary (*p<0.05*)

The concentrations of E_2_ and T were measured to determine the hormone levels at different developmental stages in both sexes. E_2_ levels in the testes did not differ at the early spermatogenesis stage, the mature stage, and the regressed stage. In the ovary, E_2_ levels showed a significant difference at the previtellogenesis stage compared with the vitellogenesis stage and the mature stage (*p<0.05*) (Fig. 9a). T levels showed significant downregulation from the mature stage to the regressed stage in the testis (*p<0.05*). There were no significant differences in T levels during ovarian development (Fig. 9b).

## Discussion

The black rockfish, as an economically crucial fish species, has been the subject of recent research in reproductive physiology, toxicology and molecular biology due to its unusual ovoviviparous reproductive strategy [28, 33, 36]. The transcriptomics profile associated with black rockfish gonadal development, however, is unclear. To investigate the mechanisms underlying gonadal development, a series of sex-related genes and biological pathways should be identified and subjected to further exploration. The present study mainly focused on the identification of the key genes and pathways in ovary and testis development and germ cell renewal by histology and RNA-seq.

### Identification of stages and terminology related to gonadal development in the black rockfish

In most teleosts, reproduction is an annual, cyclic event in which the gonads pass through different developmental stages of germ cell renewal. The critical phases of the fish reproductive cycle were determined using a conceptual model based on specific histological and physiological markers [47]. Black rockfish testes pass through a series of developmental stages, including an early spermatogenesis phase when no spermatozoa are present in the lumen of the lobules or sperm ducts, the mature phase when spermatogenesis is active, and the regressed phase in which few or no sperm are present [31, 47, 48]. In the present study, black rockfish testes in the early spermatogenesis stage, the mature stage, and the regressed stage were designated as M_III, M_IV, and M_V, respectively. In contrast to the oviparous strategy, the eggs of the black rockfish are fertilized and retained in the ovary during gestation [47]. In the present study, the identified developmental stages of the ovary were the previtellogenesis stage, the vitellogenesis stage, and the mature stage [47, 49], which were designated F_III, F_IV and F_V, respectively.

### Key pathways in testis development and gametogenesis in the male black rockfish

The testes of male black rockfish showed marked variation from the early spermatogenesis stage to the regressed stage, and this variation was reflected in both the histological results and the transcriptomic profile. During development from the early spermatogenesis stage to the mature stage, intercellular interaction and cytoskeleton pathways showed significant upregulation. Similar to mammals, cellular interactions among Sertoli cells and germ cells in the seminiferous epithelium of fish play important structural and functional roles in reproduction. The testis undergoes dramatic morpho-functional changes during the reproductive season to activate spermatogenic arrangement [50, 51]. Actin-related adhesion among Sertoli cells and Sertoli cells to germ cells is associated with multiple events that are crucial for spermatogenesis and normal fertility and especially for spermatid elongation in mature-stage testes [51, 52]. In a transcriptomics analysis of the spotted knifejaw (*Oplegnathus punctatus*), a similar intercellular interaction pathway (cell adhesion molecules) was also found to be significantly enriched in the testis [23].

In fishes, cytoplasmic extensions of Sertoli cells form an envelope around a single synchronously developing group of germ cells derived from a single spermatogonium. The formation of this group of germ cells, namely, spermatogenesis, is a highly organized and coordinated process during which diploid spermatogonia proliferate and differentiate into mature spermatozoa [2]. Both processes require large numbers of molecules, including DNA, RNA, protein, lipids, and steroids, for cell amplification. In the present study, molecular amplification, and repair in the cell cycle, including the “cell cycle”, “ubiquitin-mediated proteolysis”, “DNA replication”, “Fanconi anemia”, “mRNA surveillance”, “RNA transport”, “p53 signaling” and “apoptosis” pathways, were significantly upregulated in stage III and IV testes. In contrast to mammals, fish show a cystic type of spermatogenesis and go through different stages by an accompanying group of Sertoli cells. The shape shifting of a cyst enveloped by Sertoli cells is accompanied by strong proliferation of Sertoli cells [53, 54]. In African catfish and Nile tilapia, Sertoli cell proliferation occurs primarily during spermatogonial proliferation [54]. In *Leporinus taeniatus*, spermatogenesis is also a process in which diploid spermatogonia proliferate and differentiate into haploid spermatozoa [55]. On the other hand, the Fanconi anemia pathway has been confirmed as an efficient DNA repair pathway [56, 57]. In Fanconi anemia disease causing genes mutant zebrafish, the DNA damage repairment failed and caused a series of abnormal development.

Testis development and spermatogenesis require specific metabolites, especially androgens. In fish, transduction of the gonadotropin signal stimulates the production of 11-ketotestosterone (11-KT), a major androgen in fishes [58], functionally activating androgen receptors [59, 60]. To complete the process, enzymes and other proteins are required for biosynthesis [61]. In the present study, the steroid hormone biosynthesis pathway and the key genes *3b-hsd* and *cyp11a1* were significantly upregulated in mature-stage testes, indicating the importance of steroidogenesis for spermatogenesis [59, 62]. Similar results have also been reported in turbot (*Scophthalmus maximus*), in which steroidogenesis-related genes including *hsd17b3*, *star*, and *cyp21a2* were highly expressed in the testis during maturation [63]. Hormone level measurement during maturation in male black rockfish also indicate the importance of steroidogenesis for spermatogenesis. A few pathways related to RNA transcription and translation transportation, namely, RNA transport, mRNA surveillance pathway and ubiquitin mediated proteolysis, were also significantly enriched in mature-stage testis, suggesting that metabolism and synthesis under the influence of gonadotropins and steroid hormones is crucial for the maintenance of spermatogenesis [59, 64]. Interestingly, the pathways (progesterone-mediated oocyte maturation and oocyte meiosis) were both enriched in mature-stage testes, implying the importance of the role that progestogen plays in spermatogenesis. Serum 17α,20β-dihydroxy-4-pregnen-3-one (DHP), a fish-specific progestin, was higher at the mature stage, concomitant with testicular development in male turbot [65]. Knockout of the nuclear receptor of DHP in Nile tilapia (*Oreochromis niloticus*) resulted in a smaller testis and a lower GSI compared with normal testis [66].

### Key genes in ovarian development and gametogenesis in the female black rockfish

Due to the special reproductive strategy of the female black rockfish, the transcriptomic level changes in the ovary did not seem as marked as those in the testis. A total of 765 DEGs were significantly enriched, and these were classified into 12 categories: cell cycle, cell junction, cell structure, metabolism, DNA binding and transcription regulated, immune system, nervous system, molecular transport, protein modification, RNA binding, signal transduction and unclassified genes.

Similar to the testis of the black rockfish, ovarian development presented DEGs that were significantly enriched in pathways related to the cell cycle, cell junctions and the cytoskeleton. Matrix metalloproteinases (MMPs) are a family of extracellular proteinases that play a role in ECM remodeling associated with many physiological and pathological processes [67]. Previous studies in mammals have shown increased MMP19 expression in preovulatory follicles [68], and estrogen receptor knockout leads to downregulation of *MMP19* and failure in the release of mature oocytes [69]. In the present study, *mmp19* was also upregulated in vitellogenic ovaries, which may be related to the maturation and subsequent rupture of ovarian follicles in black rockfish.

The black rockfish *vipr* gene showed significantly increased expression in ovaries at the vitellogenesis stage, implying that it plays a critical role in folliculogenesis. The neuropeptides vasoactive intestinal polypeptide (VIP) and pituitary adenylate cyclase-activating polypeptide (PACAP) are thought to stimulate ovarian functions such as steroidogenesis and cAMP accumulation in rat granulosa cells [70] through PACAP/VIP receptors. VIPR has been found to have equal affinity for PACAP and VIP [71]. In zebrafish, *vipr* expression remained high throughout the follicle stage until the full-growth stage [72].

The classification molecular transport showed the most DEGs in ovary development of black rockfish with 20 genes of the solute carrier (SLC) gene superfamily enriched. The SLC gene superfamily encodes a series of membrane transporters [73] that includes passive transporters, cotransporters and exchangers in various cellular membranes [74]. Members of the *slc6* gene family (*slc6a11*, *slc6a15*, *slc6a6*, and *slc6a8*), which perform Na^+^- and Cl^−^-dependent transport of the neurotransmitter γ-aminobutyric acid (GABA), creatine and taurine, were differentially expressed during ovarian development. The result was coincident with the transcriptomic analysis of hapuku (*Polyprion oxygeneio*s) [75]. However, these transport proteins are more commonly studied in the central nervous system of vertebrates [76], which implies that SLCs may also function in the ovary. Zinc is necessary for meiosis in zebrafish and mammals [77, 78], and the zinc transport protein *slc30* gene family was found to be expressed in oocytes and in cumulus cells during maturation in mice (*Mus musculus*) [79], which may be a proper explanation that *slc30a9* showed up-regulated in early oocyte maturation for zinc transport. In a study on striped bass *(Morone saxatilis*), SLC family members were significantly enriched in the ovarian transcriptome, consistent with the results of the present study on black rockfish [80].

## Conclusions

The present study is the first to provide transcriptomic information on gonadal development in the ovoviviparous black rockfish. Several important candidate pathways and genes in both testis and ovarian development have been identified. Among these pathways and genes, the categories intercellular interaction and cytoskeleton, molecule amplification and repair in the cell cycle were found to be crucial in testis development and spermatogenesis along with a series of metabolite biosynthesis. Some key genes in ovarian development, such as *mmp19* and neuropeptide receptor gene *vipr*, the expression of which is important for follicle maturation and rupture, and the membrane transporter families *slc6* and *slc30*, were identified. These data provide comprehensive insight into black rockfish gonadal development and provide a basis for further study of reproductive physiology and molecular biology in ovoviviparity teleosts.

## Materials and methods

### Ethics statement

All animal experiments were reviewed and approved by the Institutional Animal Care and Use Committee of Ocean University of China. The protocol for animal care and handling used in this study was approved by the Committee on the Ethics of Animal Experiments of Ocean University of China (Permit Number: 20,141,201) prior to the initiation of the study. The studies did not involve endangered or protected species. All experiments were performed in accordance with the relevant guidelines and regulations. Briefly, individuals were anesthetized with ethyl 3-aminobenzoate methanesulfonic acid (MS-222, 0.2 g/L) and sacrificed by decapitation quickly with scissors cutting the spinal column to minimize suffering of the animals.

### Sample collection

In total, 27 adult male and 27 adult female black rockfish cultured in the northern Yellow Sea were obtained from October to March of the next year. 9 individuals were sampled for each developmental stage in all 3 developmental stages in both sexes. The fish were acclimatized at a density of 10 individuals per tank (diameter 1 m, height 1.5 m) under laboratory conditions for 2 days without feeding. After acclimation, body weight (including head) and gonad weight of each individual were measured to calculate the GSI. To ensure the status of gonad development of each fish were in the same stage, gonad of each individual was sampled and divided into 3 parts: (a) parts of gonad were fixed in Bouin’s solution for further histological analysis; (b) parts of gonad were immediately frozen in liquid nitrogen and stored at -80℃ for RNA isolation and transcriptome analysis; (c) parts of gonad were also frozen in liquid nitrogen and stored at -80℃ for further hormone concentration measurements.

### Histological analysis and RNA isolation

Part of the testes and ovaries obtained at different developmental stages were fixed in Bouin’s solution, dehydrated and embedded in paraffin. Tissue sections were cut into 6 μm by a microtome (Leica, Wetzler, Germany) and stained with hematoxylin-eosin. All photomicrographs were taken using an Olympus brightfield light microscope (Olympus, Tokyo, Japan). Other parts of the same gonad were frozen in liquid nitrogen for isolation of total RNA with TRIzol reagent (Invitrogen, USA). The quality and concentration of the total RNA were assessed by agarose gel electrophoresis and analysis on an Agilent 2100 Bioanalyzer system (Agilent Technologies, USA), respectively.

### Library construction and transcriptome sequencing

To mask the difference among sample replicates, equal amounts of total RNA from 3 individual ovaries or testes at the same developmental stage were pooled. Eighteen sequencing libraries were generated using the NEBNext® Ultra™ RNA Library Prep Kit for Illumina® (NEB, USA) according to the manufacturer’s instructions, and index codes were added to attribute sequences to each sample. The samples were sequenced on an Illumina HiSeq X Ten platform, and 150-bp paired-end reads were generated. The raw sequences have been deposited in the Short Read Archive of the National Center for Biotechnology Information (NCBI) with accession number of PRJNA573572.

### *De novo* assembly and annotation of sequencing reads

*De novo* assembly was performed on clean gonadal reads using the Trinity assembly software suite [81] without a reference genome. Transcripts (both contigs and singletons) were annotated by BLASTx searches [82] using the NCBI non-redundant (Nr), NCBI nucleotide sequences (Nt) and Swiss-Prot databases with a cutoff “e-value” of <1e^-5^. Domain-based comparisons with Protein family (Pfam) and KOG (a eukaryote-specific version of the Clusters of Eukaryotic Ortholog Groups) databases were performed by RPS-BLAST tool from locally installed NCBI BLAST + v2.2.28 and the HMMER 3.0 program, respectively. Annotated transcripts were analyzed to GO classification with the aid of the Blast2Go program [83]. These gene terms were then enriched on the three GO categories (Biological Process, Cellular Component and Molecular Function at level 2) using the GOseq R package [84]. KEGG (a database of biological systems) maps were retrieved using the online KEGG Automatic Annotation Server for the overview of metabolic pathway analysis [85, 86].

### Differential gene expression analysis

The reads obtained from each library were mapped to the *de novo* assembled transcripts using the bowtie 2 program for mismatch checking [87]. The count numbers of mapped reads and FPKM (expected number of fragments per kilobase of transcript sequence per million base pairs sequenced) were obtained and normalized by RSEM V1.2.15 [88]. Statistical analysis of differential gene expression in gonads at different developmental stages was conducted using the DEGSeq R package [89] with a cutoff “*q value*” of 0.01 and |log_2_(fold change)|>2. Transcripts with absolute fold change values greater than 2.0 were marked as significantly differentially expressed genes.

### Experimental validation by quantitative real-time PCR

To validate our Illumina sequencing data, analysis of the expression of 10 selected DEGs was performed by quantitative real-time PCR (qPCR) using specific primers. The primers used are described in Additional file 5. Samples were generated from F_III, F_IV, and F_V ovaries and from M_III, M_IV, and M_V testes. After RNA extraction and reverse transcription, all cDNA products were diluted to 500 ng/µL. The 20 µL qPCR reaction mixture consisted of 2 µL of cDNA template, 0.4 µL of each primer, 10 µL of KAPA SYBR®FAST qPCR Master Mix (2X), 0.4 µL of ROX and 6.8 µL of RNase-free water. PCR amplification was performed by incubation in a 96-well optical plate at 95 °C for 30 s followed by 40 cycles of 95 °C for 5 s, 58 °C for 30 s and a final extension at 72 °C for 2 min. qPCR was performed using the StepOne Plus Real-Time PCR System (Applied Biosystems), and the 2^−ΔΔCT^ method was used to analyze the expression level of genes.

### Measurement of hormone concentrations

Tissue homogenates were prepared from the gonads of individual fish (n=4) at different developmental stages. The tissues were ground in 0.9 % saline solution (W/V=1:9), followed by centrifugated at 3,000 rpm for 15 min at 4 °C, and then, the supernatant was stored at -80℃ for subsequent measurement. The 17β-estradiol (E_2_) and testosterone (T) levels of each individual were tested by iodine [^125^I] radioimmunoassay (RIA) kits (Beijing North, China) according to the manufacturer’s instructions. The binding rate is highly specific with low cross-reactivity to other steroids, which was less than 0.1 % for most circulating steroids.

### Statistical analysis

All data are presented as the mean values ± S.E.M. Gene expression and hormone concentrations were analyzed using one-way ANOVA followed by Duncan’s and Dunnett’s T3 multiple range tests. *p<0.05* were considered significant. All statistical analyses were performed using SPSS 19.0 software (SPSS, Chicago, IL, USA).

## Supplementary Information

**Additional file 1: Table S1.** Sampling period and related body parameters of black rockfish at different stages of gonadal development.

**Additional file 2: Table S2.** Information on the total raw reads and clean reads of 18 gonadal transcripts obtained at different developmental stages.

**Additional file 3: Table S3.** Key DEGs in each KEGG pathway identified in the testes at different developmental stages.

**Additional file 4: Table S4.** Significantly enriched DEGs in the ovaries of black rockfish at different developmental stages.

**Additional file 5: Table S5.** Primers used in quantitative real-time PCR (qPCR) validation.

## Data Availability

The datasets generated and analyzed during this study have been deposited in the Short Read Archive (SRA, http://www.ncbi.nlm.nih.gov/Traces/sra) of the National Center for Biotechnology Information (NCBI) with accession number PRJNA573572. Other data supporting the conclusion of this article are included within the article and can be found in the additional files.

## Abbreviations

DEGs: Differentially expressed genes
KEGG: Kyoto Encyclopedia of Genes and Genomes
NGS: Next-generation sequencing technology
GSI: Gonadosomatic index
GO: Gene ontology
NR: Nonredundant
BP: Biological Process
CC: Cell Component
MF: Molecular Function
ECM: Extracellular matrix
11-KT: 11-ketotestosterone
DHP: 17α, 20β-dihydroxy-4-pregnen-3-one
MMPs: Matrix metalloproteinases
vip: Vasoactive intestinal polypeptide
pacap: Pituitary adenylate cyclase-activating polypeptide
SLC: Solute carrier
GABA: γ-aminobutyric acid
NCBI: National Center for Biotechnology Information
Pfam: Protein family
KOG: Eukaryote-specific version of the Clusters of Orthologous Orthologs
